# Application of nanofat grafting to rescue a severe ischaemic hand with thromboangiitis obliterans: A case report about promising salvage procedure

**DOI:** 10.1097/MD.0000000000027577

**Published:** 2021-10-22

**Authors:** Kwan Lok Benjamin Ng, Meng-Chien Willie Hsieh, Yun-Nan Lin, Rong-Fu Chen, Tsai-Ming Lin, Sin-Daw Lin, Yur-Ren Kuo

**Affiliations:** a Department of Surgery, Kaohsiung Medical University Hospital, Kaohsiung Medical University, Kaohsiung City, Taiwan; b Division of Plastic Surgery, Department of Surgery, Kaohsiung Medical University Hospital, Kaohsiung Medical University, Kaohsiung City, Taiwan; c Charming Institute of Aesthetic and Regenerative Surgery (CIARS), Kaohsiung City, Taiwan; d Department of Surgery, Kaohsiung Municipal Siaogang Hospital, Kaohsiung City, Taiwan.

**Keywords:** case report, nanofat graft, regenerative medicine, smoking, thromboangiitis obliterans

## Abstract

**Rationale:**

Currently, there is no consensus regarding the best treatment for patients with thromboangiitis obliterans (TAO). Regenerative medicine, such as bone marrow stem cells or adipose-derived stem cell (ASC) transplantation, have proven efficacy in improving tissue perfusion and wound healing in clinical trials. In this case, we used nanofat grafting to treat severe conditions in a patient with TAO, with promising outcomes.

**Patient concerns:**

This is a case of a 48-year-old smoker who presented with cyanosis in both hands and the right foot, with gangrenous changes. Investigative angiography showed severe vasospasm in the radial and ulnar arteries of the patient's left hand. Progressive cyanosis of the patient's left hand was noted which may eventually require amputation if left untreated.

**Diagnoses:**

He was diagnosed with TAO under the Shionoya diagnostic criteria.

**Interventions:**

Fasciotomy and necrotic tissue debridement were performed, followed by centrifuged nanofat grafting. The nanofat graft was prepared using Pallua method and deployed with a MAFT-GUN (Dermato Plastica Beauty Co., Ltd., Kaohsiung, Taiwan).

**Outcomes:**

Three months later, computed tomography angiography revealed a radial artery patency. The patient's wrist function was preserved with uneventful wound healing.

**Lessons:**

The regenerative ability of centrifuged nanofat grafts not only helps wound healing but also helps reverse vasospasm and preserve remnant tissue perfusion.

## 1 Introduction

Thromboangiitis obliterans (TAO), or Buerger disease, is an uncommon nonatherosclerotic segmental inflammatory vasculitis that usually affects the small and medium arteries of the hands and feet of male smokers, with a typical age range of 2 to 50 years. The aetiology and mechanism of TAO remain unknown.^[1]^ Immunological dysfunction, tobacco hypersensitivity and endothelial dysfunction^[2]^ may play an important role in the pathogenesis of TAO.^[3]^

Currently, there is no consensus about the best treatment for patients with TAO.^[4]^ Regenerative medicine, such as bone marrow stem cell (BMSC)^[5]^ or adipose-derived stem cell (ASC) transplantation,^[6]^ has proven efficacy in improving tissue perfusion and wound healing in clinical trials.^[7]^ Procedure of nanofat grafting, first described by Tonnard et al,^[8]^ allows quick isolation of ASCs from lipoaspirate. Due to its regenerative abilities, nanofat is widely used clinically in the fields of wound healing and skin rejuvenation.^[9–12]^

Fat extract has been proven to attenuate ischemic injury and stimulate angiogenesis in ischemic tissues.^[13]^ In this case, we used nanofat grafting to treat severe conditions in a patient with TAO, with promising outcomes.

## 2 Case report

**Figure 2:**
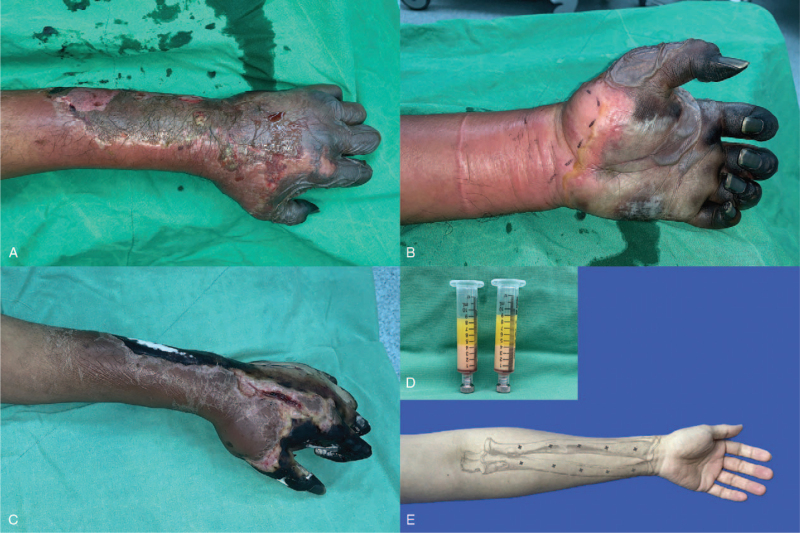
Surgical intervention of the left forearm in the acute phase. A: Anterior view of the left forearm before debridement. B: Posterior view of the left forearm before debridement. C: Anterior view of the left forearm before fat grafting. D: A total of 10 mL centrifuged nanofat was prepared before emulsification. E: Intramuscular injection sites were marked along the radius and ulna. Progressive tissue necrosis over the left forearm is illustrated in panels A and B. After discarding the oil and liquid layers of the centrifuged native graft, the remnant centrifuged native fat was emulsified and administered intramuscularly along the marks as indicated. (× site of fat injection).

A 48-year-old man with mood disorder under medication and with a smoking history of two packs of cigarettes per day for over 30 years presented to our internal medicine outpatient department because of cyanosis in both hands and the right foot, with severe pain. Physical examination showed that the left hand was cold and had an absent pulse in the left radial artery. The patient was hospitalised for treatment. Urokinase and prostaglandins were prescribed, and a radiologist was consulted for investigative angiography. Left upper limb angiography revealed the total absence of blood flow in the radial and ulnar arteries (Fig. 1A), with no evidence of atherosclerosis or thromboembolism. The involvement of small- and medium-sized vessels with the most severe type of vascular atresia was also noted. Owing to the above clinical course, Shionoya diagnostic criteria were fulfilled, and TAO was suggested.^[14]^ due to the progression of cyanosis despite the use of the aforementioned medications, a cardiovascular specialist was consulted for percutaneous transluminal angioplasty, but the examination failed due to severe vasospasm. A plastic surgeon was consulted for the surgical intervention. Preoperative physical examinations showed necrotic tissues within the left hand (Fig. 2A and 2B), while laboratory data showed elevated inflammatory parameters, with high C-reactive protein levels up to 249 mg/L. Fasciotomy with debridement was performed smoothly (Fig. 2C). The wound was then dressed with Biodyne ointment (China Chemical & Pharmaceutical Co., Ltd., Taiwan) and AQUACEL Foam (ConvaTec Inc., NC).

**Figure:**
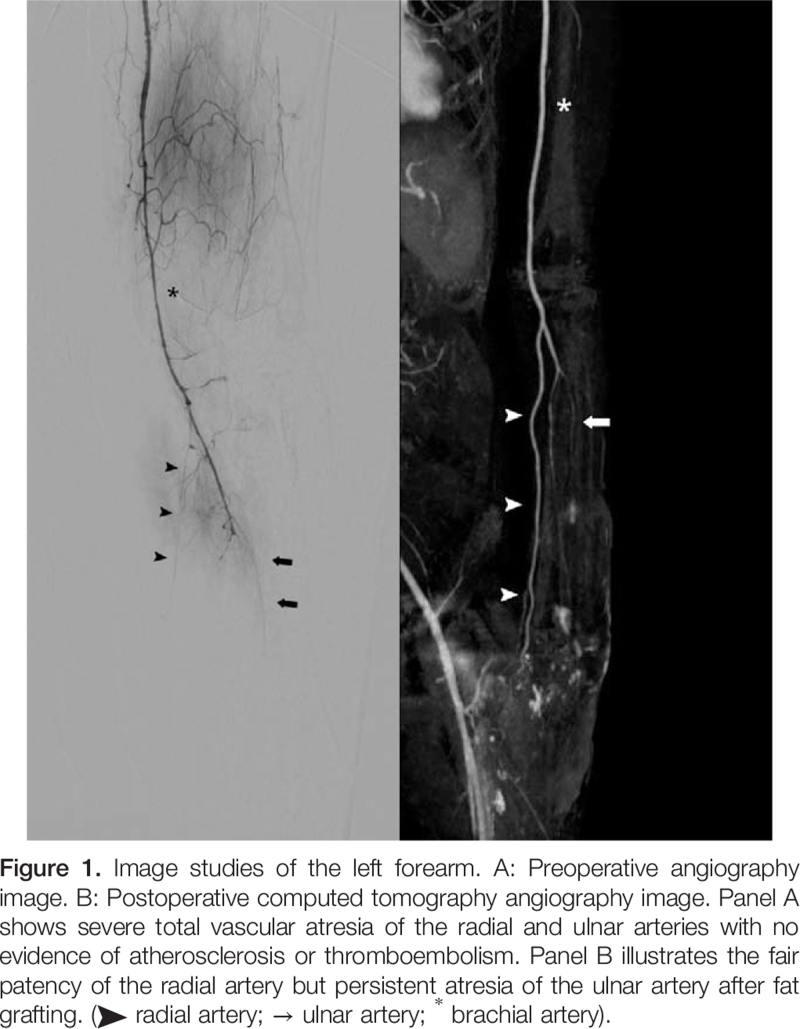


Nine days after the first operation, fat grafting has been suggested as an alternative treatment for this critical condition. Informed consent was obtained and fat grafting was performed. The operation technique was performed using Pallua et al^[15]^ method. A tumescent solution was administered at the lower abdomen donor wound; the fat was harvested using a blunt-tip suction cannula (diameter 2.5 mm; with three elliptical side holes). The lipoaspirate volume approximating the amount of the administered tumescent solution was obtained to ensure a high ratio of native fat after processing by centrifugation. Low pressure was employed, and the extracted native fat was processed and concentrated by centrifugation at 1200 × *g* for 3 min. The oil on top of the syringe was wiped, leaving only the middle portion containing centrifuged native fat to be used for the following preparation (Fig. 2D and 2E). The centrifuged native fat was then mechanically emulsified by shifting the fat between two 10-cc syringes connected to each other by a female-to-female Luer-Lok connector. Subsequently, centrifuged nanofat was transferred from the 10-mL syringe to a 1-mL syringe by connecting the two syringes using a syringe transducer. The centrifuged nanofat-filled syringe was then loaded into a MAFT-GUN (Dermato Plastica Beauty Co., Ltd., Kaohsiung, Taiwan).^[10]^ The fat parcel volume administered by each trigger was set by adjusting a six-graded dial to control the total injection aliquot per 1 mL of fat graft. A 16 G blunt cannula was used to administer the fat while withdrawing MAFT-GUN. Each delivered fat parcel was set at 1/60 mL (each parcel volume, 0.017 mL) and was dispersed throughout his left forearm from the proximal to distal end along both the radial and ulnar sides through intramuscular injection. The sites of injection were as indicated (Fig. 2E), with 1 mL of fat graft per injection (a total of 10 mL fat graft). After the operation, the injection sites were covered with gauze, while the previously debrided wound was dressed with Biodyne ointment and AQUACEL Foam.

**Figure 3:**
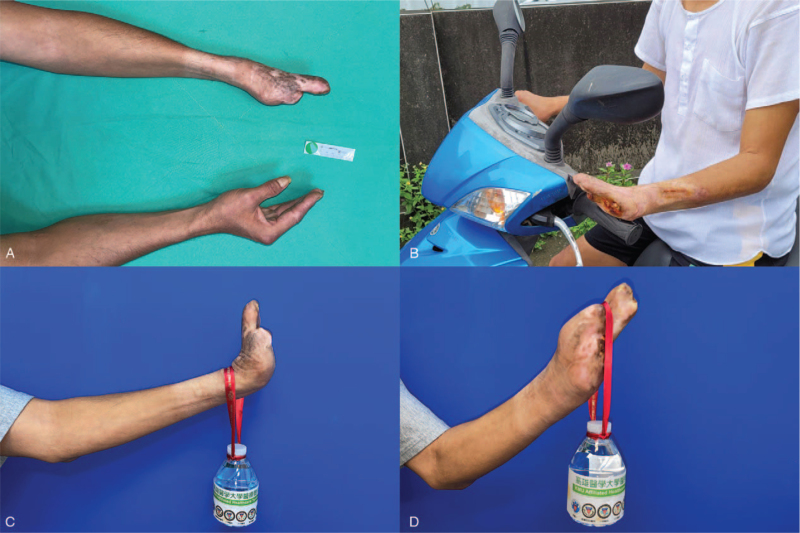
Postoperative images and functional demonstration of left-hand amputation stump. A: Neutral positions of the bilateral forearms. B: Fair control of motorcycle steering handle with bilateral hands. C: Preserved wrist extension function. D: Slinging the water bottle on the concave of the left hand. A follow-up image of the successfully preserved wrist function at 20 months was obtained. The patient was grateful for preserving his wrist function, because this was helpful for him to hang a bag and push an object in his daily life. He could even travel by motorcycle on his own.

The patient was discharged after 3 days of intravenous antibiotic treatment and was under close surveillance at the plastic surgery outpatient department. The patient was re-admitted 82 days after centrifuged nanofat grafting, and a preoperative survey of three-dimensional computed tomography angiography revealed the patency of the radial artery of the left hand which indicated the success of the treatment (Fig. 1B). The amputation of the left 1^st^ to 5^th^ fingers and 4^th^ to 5^th^ metacarpals, debridement of necrotic tissues, and reconstruction of tissue defects with skin grafts were performed. After the operation, his wrist function was preserved with uneventful wound healing without complaints of rest pain (Fig. 3A, 3C, and 3D).

## 3 Discussion

Patients with TAO may have specific cellular immunity against arterial antigens, specific humoral anti-arterial antibodies, and elevated circulatory immune complexes.^[16]^ These antibodies and immune complexes are believed to be the main cause of vascular endothelial dysfunction. Eichhorn et al^[17]^ showed that anti-endothelial cell antibody was elevated in patients with TAO and that the antibody titers were proportional to the disease severity. Makita et al^[18]^ demonstrated impaired endothelium-dependent vasorelaxation in the peripheral vessels of patients with TAO. Flow-mediated vasodilation, an index of endothelium-dependent vasodilation, was shown to be significantly smaller in the TAO group than in the control group.^[19]^ Tissue perfusion throughout the distal limbs of patients with TAO is compromised, and one of the many reasons behind this is vasospasm. This impairment eventually leads to ischemia, poor wound healing, and pain.

Yamamoto et al^[20]^ examined sympathetic tone in patients with TAO by measuring their muscle sympathetic nervous activity. Their data showed that patients with TAO had a higher sympathetic responsiveness than the control group. This sympathetic overactivity increases vasoconstrictor activity and vascular resistance, which might contribute to the pathophysiology of arterial vasospasm and explain the effectiveness of sympathectomy. Another possible pathophysiologic mechanism of vasospasm in patients with TAO might be local inflammation of tissues, which in turn leads to an increased sympathetic response and causes vasoconstriction. The anti-inflammatory effects of ASCs help suppress local inflammation and explain the vasospasm-relieving effect of ASCs.^[21]^

With regenerative ability, ASCs are becoming increasingly popular in treating recalcitrant wounds and critical limb ischemia.^[5,10,22,23]^ The mechanisms of autologous ASC implantation in treating TAO are multifold. First and foremost, ASCs secrete factors such as interleukin-10 and cytokines that can attenuate inflammation and help shorten the inflammatory status.^[21]^ These growth factors may help improve tissue perfusion and exert anti-inflammatory effects in patients with TAO. Second, in the treatment of TAO, ASCs consist of angiogenic factors that can help promote angiogenesis, enhance wound healing and improve limb ischaemia.^[6]^ The implantation of ASCs or BMSCs has been proven to be effective in the treatment of TAO.^[5,6,23]^ Tonnard et al^[8]^ first described the production process of ASCs using emulsification. The viability evaluation shows that the normal fat tissue structure of the nanofat is lost after emulsification and that adipocytes are eliminated during this physical processing, with ASCs remaining in the emulsified nanofat.^[8,24]^ Recently, Pallua et al demonstrated that centrifuged nanofat still had adequate cell numbers of ASCs and endothelial progenitor cells and with fair CD73, CD90, and CD105 expression in ASCs and endothelial progenitor cells. In this case, we chose to use Pallua et al protocol to condense the injected volume. These nanofat grafts can be harvested easily in the operating room without the need for stem cell culture in the laboratory. This simple harvesting method acts as an alternative practical way to relieve patients with TAO due to severe vascular spasm in the acute phase.

Ilenia et al described a 29-year-old patient with a smoking history who presented with wet gangrene, necrosis of the fifth toe, and extensive plantar ulcerations on both feet. The patient was diagnosed with TAO. Amputation of the gangrenous toe was performed, but poor healing of the surgical wound was noted. Centrifuged native fat grafts were then harvested and inoculated 1 cm above the end of the terminal arteries. The dehiscent wound was almost healed at 60 days post procedure.^[9]^ Kim et al^[25]^ described that 72% (16 of 22 limbs) of autologous BMSC implantations in patients with TAO had angiogenesis with the growth of collateral vessels into distal lower limbs. Interestingly, in our case with centrifuged nanofat grafts as the source of ASCs, not only did the wound heal well, but the patency of previously occluded vessels was also noted. The vasospasm of our patient's radial artery improved after centrifuged nanofat grafting. This procedure helped lower the level of amputation, preserve the wrist function of this patient, and improve his quality of life (Fig. 3B).

However, the pathophysiology of TAO and the mechanisms of fat grafting in patients with TAO are still under investigation. The standardisation of therapeutic protocols and the dosage required for this therapy remain uncertain before wide clinical application in cases similar to ours. Moreover, we could not compare the effects of medications alone with fat grafting on patients with TAO; therefore, additional case–control studies are required for comparison. Despite the limitations mentioned above, our results indicate the benefits of this simple procedure in patients with TAO in the acute phase.

## 4 Conclusion

Autologous centrifuged nanofat grafting is an alternative regenerative medicine technique used to rescue the gangrenous hand of this patient with TAO in the acute phase. To the best of our knowledge, this case report shows that the regenerative ability of centrifuged nanofat grafts not only helps wound healing but also helps reverse vasospasm and preserves remnant tissue perfusion.

## Author contributions

**Conceptualization:** Yun-Nan Lin.

**Resources:** Tsai-Ming Lin, Yur-Ren Kuo.

**Writing – review & editing:** Kwan Lok Benjamin Ng, Yun-Nan Lin, Meng-Chien Willie Hsieh, Rong-Fu Chen, Sin-Daw Lin, Yur-Ren Kuo.
